# Factor structure and internal reliability of an exercise health belief model scale in a Mexican population

**DOI:** 10.1186/s12889-017-4150-x

**Published:** 2017-03-01

**Authors:** Oscar Armando Esparza-Del Villar, Priscila Montañez-Alvarado, Marisela Gutiérrez-Vega, Irene Concepción Carrillo-Saucedo, Gloria Margarita Gurrola-Peña, Norma Alicia Ruvalcaba-Romero, María Dolores García-Sánchez, Sergio Gabriel Ochoa-Alcaraz

**Affiliations:** 1 Psychology Program, Universidad Autónoma de Ciudad Juárez, Av. Universidad y H. Colegio Militar s/n, Ciudad Juárez, Chihuahua Mexico; 2 Facultad de Ciencias de la Conducta, Universidad Autónoma del Estado de México, Carretera Toluca - Naucalpan km. 1.5 s/n, Toluca, Estado de México Mexico; 3 Centro Universitario de Ciencias de la Salud, Universidad de Guadalajara, Sierra Mojada N° 950. Col. Independencia, C.P. 44350 Guadalajara, Jalisco Mexico; 4 Unidad Académica de Psicología, Universidad Autónoma de Zacatecas, Av. Preparatoria 301, Hidraulica, 98060 Zacatecas, Zacatecas Mexico; 5 Facultad de Psicología, Universidad de Colima, Av. Universidad No. 333, Las Víboras, CP 28040 Colima, Colima Mexico; 6 Instituto de Ciencias Sociales y Administración, Universidad Autónoma de Ciudad Juárez, Av. Universidad y H. Colegio Militar s/n, Zona Chamizal, Ciudad Juárez, Chihuahua Mexico

**Keywords:** Exploratory factor analysis, Physical activity, Health models, Overweight, Obesity

## Abstract

**Background:**

Mexico is one of the countries with the highest rates of overweight and obesity around the world, with 68.8% of men and 73% of women reporting both. This is a public health problem since there are several health related consequences of not exercising, like having cardiovascular diseases or some types of cancers. All of these problems can be prevented by promoting exercise, so it is important to evaluate models of health behaviors to achieve this goal. Among several models the Health Belief Model is one of the most studied models to promote health related behaviors. This study validates the first exercise scale based on the Health Belief Model (HBM) in Mexicans with the objective of studying and analyzing this model in Mexico.

**Methods:**

Items for the scale called the Exercise Health Belief Model Scale (EHBMS) were developed by a health research team, then the items were applied to a sample of 746 participants, male and female, from five cities in Mexico. The factor structure of the items was analyzed with an exploratory factor analysis and the internal reliability with Cronbach’s alpha.

**Results:**

The exploratory factor analysis reported the expected factor structure based in the HBM. The KMO index (0.92) and the Barlett’s sphericity test (*p* < 0.01) indicated an adequate and normally distributed sample. Items had adequate factor loadings, ranging from 0.31 to 0.92, and the internal consistencies of the factors were also acceptable, with alpha values ranging from 0.67 to 0.91.

**Conclusions:**

The EHBMS is a validated scale that can be used to measure exercise based on the HBM in Mexican populations.

## Background

In 2014, approximately 1.3 billion of adults worldwide, that were 18 years old and older, were overweight and 600 million were obese [1]. In many countries, being overweight or having obesity kills more people than being underweight [1]. In Mexico, 42% of men were overweight and 26.8% of men were obese, while 35.5% of women were overweight and 37.5% of women were obese [2]. Health risks related to overweight and obesity include cardiovascular diseases (leading cause of death in 2012), diabetes, musculoskeletal disorders and some cancers [1]. Exercise can help prevent, slow down the progression, or manage these diseases associated with overweight and obesity [3–8]. There are also several studies, including meta-analyses, that have found exercise interventions to be effective in reducing weight and body mass index in people with overweight or obesity [9–14].

Obesity and overweight are a public health problem in Mexico that needs to be attended to and it is important that this problem is dealt by analyzing and evaluating models that shape and change health behaviors. There are numerous psychosocial models that study and explain behavioral change in health. The World Health Organization (WHO) summarizes the most effective models and theories of health promotion and education that have been effective in practice including the Rational Model, Extended Parallel Process Model, Transtheoretical Model of Change, Theory of Planned Behavior, Activated Health Education Model, Social Cognitive Theory, Communication Theory, Diffusion of Innovation Theory, and the Health Belief Model [15]. These models and theories have been involved in the promotion of health behaviors by enabling people to increase control over and to improve their health [15]. From these models and theories, the Health Beliefs Model (HBM) has been shown to explain changes in people’s health behaviors [16], including exercise [17–20]. There are several studies that apply the HBM to physical activity [21–23], but physical activity and exercise are defined differently. Physical activity is “any bodily movement produced by skeletal muscles that results in energy expenditure… [and] in daily life can be categorized into occupational, sports, conditioning, household, or other activities” [24], and exercise is “a subset of physical activity that is planned, structured, and repetitive and has a final or an intermediate objective, the improvement of physical fitness” [24]. Nevertheless, some studies use the term physical activity and exercise interchangeably [24].

### History of the health belief model

Over time people have been concerned about health. It is for this reason that professionals committed to this area have conducted research and interventions, and have also developed theories and models that explain health behaviors of individuals [16, 25]. In the 50s, in an effort to build a psychosocial model to explain behaviors related to health and prevention, the conceptual basis of the Health Belief Model [26–28] was formulated in collaboration with Mayhew Derryberry, creator of the Division of Behavior Studies in the Department of Public Health of the United States of America and of a group of four social psychologists: Godfrey Hochbaum, Stephen Kegeles, Hugh Leventhal and Irwin Rosenstock [16, 29].

The studies conducted by Hochbaum, in 1952, related to a prevention program against tuberculosis, were fundamental for the development of the HBM [30]. These studies observed more than 1200 adults in three American cities and their willingness to undergo X-ray examinations. They found that their willingness to undergo examinations was the product of individual beliefs of susceptibility to the disease and personal benefits of early detection [30]. The HBM was proposed, at first, to give an explanation and prediction of preventative behaviors and to know the reasons for people not going to medical examinations for early detection of diseases or simply to know their health status, among others preventive behaviors. The HBM began to arouse interest in different professionals from different countries in such a manner that by the 1970′s, the model began to be used in research and health interventions, and evidence in favor of the model began to be published [25]. In 1984, Janz and Becker [31] examined the HBM to account for its effectiveness in practice by reviewing 46 studies that included health behaviors like breast self-exams, vaccinations, exercise, physical activity, smoking, seat belt use, among others. Currently, the HBM is considered useful [25], and valid [29] as it is one of the most applied models in the promotion of health [32] and has been one of the most cited and used models to explain behaviors related to disease prevention, symptom responses, and diseases as well as other behaviors with health effects [16].

### Factor structure of the health beliefs model

The HBM is based on three main assumptions: 1) the belief that a problem is extremely important to take it into account, 2) the perception of vulnerability because of that problem and, 3) the perception that the action taken will have, as result, a greater benefit compared with the personal cost produced. According to this model, the interaction of these assumptions stimulates the appearance of healthier behavior patterns that allow to prevent diseases and avoid risky situations [32]. The HBM is composed of two factors that explain health behaviors: the perception of health threat and the perception that specific health behavior can reduce or eradicate the threat [33].

**Fig. 1:**
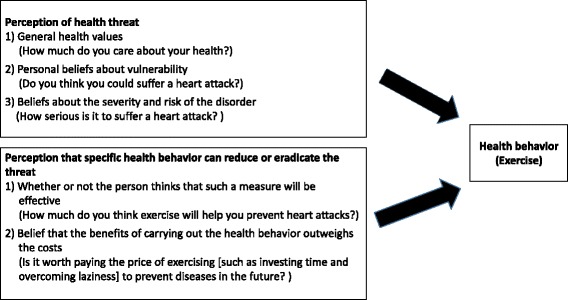
Health belief model applied to exercise

The perception of health threat has three components (Fig. 1): 1) general health values, which refer to the interest and concern for one’s health, 2) personal beliefs about vulnerability and 3) beliefs about the severity and risk of the disorder. For example, people can change their behavior and start exercising if, 1) they are really concerned about their health, 2) they believe that by not exercising they can suffer from some illnesses and, 3) that suffering from those illnesses is very serious leading to a low quality of life or death [33].

The reduction or disappearance of the perceived threat by adopting a health behavior has two components: 1) whether or not the person thinks that such a measure will be effective and, 2) the belief that the benefits of carrying out the health behavior outweigh the costs. For example, a person who does not exercise, feels vulnerable about suffering from related illnesses, and maybe thinking about starting to exercise, may think that 1) exercising reduces the risk or illnesses, and 2) although it is hard to exercise, the ultimate benefit will be better than the potential harm to health, so the person will decide to modify their behavior [33].

The HBM has been applied and studied in several health behaviors [34] like tuberculosis treatment adherence [35], breast self-examination [36], osteoporosis prevention [37], cervical cancer screening [38], hepatitis A and B vaccination [39], Pap smear testing [40], Human Papilloma Virus vaccination [41], prostate cancer screening [42], colorectal cancer screening [43], high-risk sexual behaviors [44], physical activity [22, 23, 45], and exercise [17–20] among others with good results in explaining health behaviors.

There are several exercise studies using the HBM around the world where positive outcomes have been found. In a study done with a sample of 98 Jordanian myocardial infarction patients [17], with a mean age of 50 years (SD = 12.15), and 58% males, it was found that health motivation and perceived barriers had statistically significant correlations with exercise participation. A study with a sample of 132 Hong Kong adults [18], with a mean age of 49.3 years (SD = 9.46), and 59.8% female, found a statistically significant standardized beta, in a multiple regression analysis, between exercise and perceived barriers. In a different study with a sample of 57 participants from New York [19], with a mean age of 56 years (SD = 10.4) and 72% males, the HBM accounted for 29% of the variance of exercise attendance, and three HBM factors were associated with exercise attendance: perceived severity of coronary heart disease, perceived benefits of exercise, and special health practices. Currently there are no published articles that have studied exercise using the HBM in Mexico.

For this reason, the HBM will be used to explain exercise behavior in the Mexican population. Several articles that have studied the HBM design and use their own scales and for many of those studies the validation is not reported. There are some scales that have been validated, like the Champion’s Health Belief Model Scale for breast cancer [46], but a few studies use it. Exercise and physical activity studies, based on the HBM, have no validated scales used across studies, and most of them develop their own instruments without validating the scales. One of the studies used a scale, the HBM Questionnaire [47], but the original scale was designed for dieting and fasting and the study does not show the validation of the modified scale. Also, each factor was composed of only one item, when most of the other scales use more items per factor. There are no validated exercise scales based on the HBM in Spanish, so the purpose of this study was to develop and validate a scale by developing items, analyzing the factor structure, and analyzing the internal reliability of the factors. The validation of this scale will help researchers study exercise using the HBM in Mexico.

## Method

### Participants

The sample consisted of 746 participants from five cities of Mexico: Juarez (23.1%), Zacatecas (22.6%), Toluca (22.2%), Colima (17.5%) and Guadalajara (14.5%). The mean age for the total sample was 28.54 years (SD = 18.89) and 54.6% reported being female and 44.1% male. With respect to marital status, 57.7% reported being single, 34.1% married, 4.4% living common law, 2.0% widowed, 1.0% divorced, and 0.8% separated. Demographic information is described in Table 1.

**Table 1:** Demographic information by city

	Juarez	Guadalajara	Toluca	Colima	Zacatecas	F or *Χ* ^2^ (p)	Post Hoc (Bonferroni)
N	170	104	168	121	167		
Mean Age (SD)	33.11 (29.68)	34.18 (14.46)	24.00 (12.36)	18.99 (6.93)	32.29 (14.81)	19.12 (<0.01)	J > T; J > C; G > T; G > C; Z > T; Z > C
Gender							
Female (%)	52.9	58.7	56.0	47.9	60.5	5.35 (0.25)	
Male (%)	47.1	41.3	44.0	52.1	39.5		
Marital Status							
Married (%)	50.0	46.7	20.0	7.8	43.7	165.12 (<0.01)	
Single (%)	44.7	46.7	74.3	90.6	36.7		
Divorced (%)	0.6	2.8	0.0	0.0	1.8		
Separated (%)	0.6	0.0	1.3	0.8	1.2		
Living Common Law (%)	3.5	1.9	1.3	0.0	13.0		
Widowed (%)	0.6	1.9	3.1	0.8	3.6		

Note: *J* Juarez, *G* Guadalajara, *T* Toluca, *C* Colima, *Z* Zacatecas

MacCallum and colleagues criticize the traditional rules of thumb commonly used to calculate the necessary sample sizes to perform exploratory factor analyses that recommend a specific number or participants per item [48]. In their study they determine that sample size depends on three indicators: item communalities, number of factors, and the number of items per factor. MacCallum and colleagues suggest that “If results show a relatively small number of factors and moderate to high communalities, then the investigator can be confident that obtained factors represent a close match to population factors, even with moderate to small sample sizes” [48]. The sample size for the exploratory factor analysis is sufficient since the range of the item communalities were between moderate and high, there were a few factors, and there were several items per factor.

### Instruments

#### Exercise health belief model scale (EHBMS)

This scale consisted of 32 items with five-point Liker-type response option scale: for items one to 26 the options were “not at all”, “a little”, “more or less”, “quite a bit”, and “a lot” due to the type of items, for example “How much interest do you have for your health” and “Is it worth paying the price of exercising [such as investing time and overcoming laziness] to prevent diseases in the future?”; for items 27 to 32 the response options were “I don’t believe”, “maybe, but it’s unlikely”, “I believe it’s likely”, “I believe it’s very likely”, and “I believe, I’m sure of it” because these items are different from the previous ones since they ask if they think they can get diseases, for example “Do you think you could get high blood pressure”. After the exploratory factor analysis, the items divided into five factors: general health values, beliefs about the vulnerability of not exercising, beliefs about the severity of not exercising, beliefs that exercising can reduce threats, and beliefs that the benefits exceed the costs of exercising. The internal reliability of each factor, analyzed with Cronbach’s alpha, was 0.84, 0.67, 0.90, 0.85 and 0.75, respectively.

#### Socio-demographic questionnaire

Participants were asked to report their age, gender, and marital status.

### Procedure

The study was evaluated and approved by the ethics committee of the Autonomous University of Juarez. Items were written for each of the five factors of the Health Belief Model by a research group composed of five researchers with doctoral degree with expertise in health behavior. A total of 52 items were written for the scale in the first draft. Then, to evaluate the easiness of reading, response options and the redaction of the items, the scale was administered to a convenience sample of 50 participants, who were asked to evaluate their content and meaning. A sample of 10 participants per city was obtained from different areas. Researchers went to different neighborhoods to ask people to participate. Participants read each item, they were asked if they understood the items, to explain the meaning of the items, to suggest any changes if the item was not clear, and to write what part of the item they did not understand if it was the case. At the end, a researcher would talk to the participants to gather more information if needed. According to their suggestions, some items were eliminated and others were modified based on the opinions of the sample having as a result 32 items representing the five factors of the HBM. The final scale was applied to a sample of 746 participants from the cities of Juarez, Zacatecas, Toluca, Colima y Guadalajara.

Participants were given a written consent form that included the description of the study, ethical issues of the study and their participation. Participants confidentiality was ensured by not asking for identifying information, and they were given time to ask for more information about the study if required before administering the scales. The scales were given to participants and it took them between 10 and 15 min to answer. As part of the instructions on the scale, participants were given the definition of exercise and it was explained how it was different from physical activity. Data was entered and analyzed in SPSS 24 with exploratory factor analysis for the scale, and demographic information was analyzed with analysis of variance and chi square analyses.

## Results

### Elaboration of the items

Items were written in Spanish and revised by a group composed of five researchers with doctoral degrees with expertise in health topics. The group wrote items based on the HBM model and made a pool including all of them. They revised each of the items and chose the most representative of each factor. The items were administered to a sample of 50 participants who were asked specifically if they understood all the words, the easiness of reading, the response options, and suggestions if they did not understand the item. A final list of 32 items was considered to represent the factors of the scale with the response options previously described (see Table 2).

**Table 2:** Exploratory factor analysis of the health belief model questionnaire

	Factor loadings					
Item	1	2	3	4	5	h^2^
1. How much interest do you have for your health? (¿Qué tanto interés tienes por tu salud?)	0.49	0.48	-0.05	0.41	**0.85**	0.75
2. How much do you think about your health? (¿Qué tanto piensas acerca de tu salud?)	0.42	0.44	0.02	0.34	**0.79**	0.65
3. How much do you care about your health? (¿Qué tanto te preocupa tu salud?)	0.48	0.47	0.03	0.39	**0.82**	0.70
4. How important do you think is taking care of your health? (¿Qué tan importante crees que es cuidar de tu salud?)	0.25	0.24	0.10	0.24	0.28	0.11
5. How serious is it to suffer from high blood pressure? (¿Qué tan grave es padecer de presión alta?)	0.22	0.24	0.14	0.33	0.21	0.14
6. How serious is it to have diabetes? (¿Qué tan grave es padecer de diabetes?)	0.14	0.21	0.02	0.31	0.15	0.15
7. How serious is it to suffer a heart attack? (¿Qué tan grave es que te dé un infarto al corazón?)	0.18	0.20	0.03	**0.45**	0.20	0.32
8. How serious is it to suffer a stroke? (¿Qué tan grave es que te dé una embolia?)	0.47	0.46	0.11	**0.91**	0.38	0.84
9. How serious is it to get cancer? (¿Qué tan grave es que te dé cáncer?)	0.47	0.47	0.09	**0.85**	0.37	0.74
10. How serious is it to gain weight? (¿Qué tan grave es que aumentes de peso?)	0.18	0.16	0.15	0.29	0.16	0.19
11. How much do you think exercise will help you prevent having high blood pressure? (¿Qué tanto crees que el ejercicio te ayude a prevenir tener presión alta?)	0.51	**0.79**	0.13	0.47	0.45	0.70
12. How much do you think exercise will help you prevent [or control] diabetes? (¿Qué tanto crees que el ejercicio te ayude a prevenir [o controlar] la diabetes?)	0.52	**0.87**	0.17	0.44	0.43	0.80
13. How much do you think exercise will help you prevent heart attacks? (¿Qué tanto crees que el ejercicio te ayude a prevenir los infartos al corazón?)	0.54	**0.84**	0.09	0.45	0.46	0.74
14. How much do you think exercise will help you prevent strokes? (¿Qué tanto crees que el ejercicio te ayude a prevenir las embolias?)	0.49	**0.82**	0.14	0.38	0.43	0.74
15. How much do you think exercise will help you prevent cancer? (¿Qué tanto crees que el ejercicio te ayude a prevenir el cáncer?)	0.37	**0.68**	0.10	0.31	0.37	0.55
16. How much do you think exercise will help you not to gain weight? (¿Qué tanto crees que el ejercicio te ayude a no aumentar de peso?)	0.59	**0.71**	0.08	0.47	0.47	0.65
17. How much do you think exercise will help you have better health? (¿Qué tanto crees que el ejercicio te ayude a tener una mejor salud?)	0.64	**0.74**	0.02	0.51	0.53	0.73
18. How much do you think exercise will help you have a better quality of life? (¿Qué tanto crees que el ejercicio te ayude a tener una mejor calidad de vida?)	0.66	0.75	0.04	0.48	0.55	0.71
19. How much do you think exercise helps you live longer? (¿Qué tanto crees que el ejercicio te ayude a vivir más años?)	0.10	0.14	-0.02	0.10	0.13	0.16
20. How much do you think exercise will help you look better? (¿Qué tanto crees que el ejercicio te ayude a verte mejor?)	0.73	0.55	0.07	0.44	0.49	0.64
21. Is it worth paying the price of exercising [such as investing time and overcoming laziness] to prevent diseases in the future? (¿Vale la pena pagar el precio de hacer ejercicio [como invertir tiempo y superar la flojera] para prevenir enfermedades en un futuro?)	**0.41**	0.24	0.04	0.22	0.23	0.19
22. Is it worth paying the price of exercising [such as investing time and overcoming laziness] to have better health? (¿Vale la pena pagar el precio de hacer ejercicio [como invertir tiempo y superar la flojera] para tener una mejor salud?)	**0.91**	0.52	0.13	0.44	0.45	0.85
23. Is it worth paying the price of exercising [such as investing time and overcoming laziness] to have a better quality of life? (¿Vale la pena pagar el precio de hacer ejercicio [como invertir tiempo y superar la flojera] para tener una mejor calidad de vida?)	**0.92**	0.56	0.15	0.45	0.50	0.87
24. Is it worth paying the price of exercising [such as investing time and overcoming laziness] to live longer? (¿Vale la pena pagar el precio de hacer ejercicio [como invertir tiempo y superar la flojera] para vivir más años?)	**0.86**	0.53	0.12	0.41	0.46	0.79
25. Is it worth paying the price of exercising [such as investing time and overcoming laziness] to look better? (¿Vale la pena pagar el precio de hacer ejercicio [como invertir tiempo y superar la flojera] para verte mejor?)	**0.81**	0.54	0.12	0.39	0.45	0.71
26. Even if I find it hard to exercise, it is worth doing to prevent diseases in the future. (Aunque me cueste hacer ejercicio, vale la pena hacerlo para prevenir enfermedades en un futuro.)	**0.74**	0.49	0.16	0.41	0.40	0.64
27. Do you think you could get high blood pressure? (¿Crees que te pueda dar presión alta?)	0.19	0.14	**0.81**	0.19	0.09	0.70
28. Do you think could get diabetes? (¿Crees que te puede dar diabetes?)	0.13	0.13	**0.82**	0.11	-0.02	0.70
29. Do you think you could suffer a heart attack? (¿Crees que puedes tener un infarto al corazón?)	0.08	0.08	**0.82**	0.09	-0.01	0.69
30. Do you think you could suffer a stroke? (¿Crees que te puede dar una embolia?)	0.05	0.06	**0.41**	0.05	0.01	0.22
31. Do you think could get cancer? (¿Crees que te puede dar cáncer?)	0.09	0.11	**0.66**	0.06	0.01	0.50
32. Do you think you could gain weight? (¿Crees que puedes aumentar de peso?)	0.15	0.11	**0.61**	0.14	0.00	0.49

Note: Highest loadings are in bold; The items were developed and applied in Spanish, and were only translated to English for this table

### Exploratory factor analysis

The 32 items were analyzed with an exploratory factor analysis (EFA) by constraining the structure to the five factors as proposed by the model, using the generalized least squares method with a promax oblique rotation. The factor loadings for most of the items had their highest loading in the expected factors (see Table 2). The KMO index [49] was 0.92 and the Barlett’s sphericity test [49] was statistically significant (*p* < 0.01) indicating an adequate sample and normal distribution for the EFA. Factor loadings were analyzed and an item was retained in a factor if the highest loading had a value of 0.30 or greater and if the difference of the highest loading and the second highest loading was at least 0.10. If the highest factor loading of an item was less than 0.30, the item was excluded, and if an item had shared factor loadings in two or more factors (factor loadings difference less than 0.10), then the item was also excluded.

The final scale had 25 items with unique factor loadings ranging from 0.31 to 0.92 (see Table 2), three items were excluded because of low factor loading values (items 4, 10, and 19), two items were excluded because of shared factor loadings (items 5 and 18), one item was excluded because it had the highest loading in a different factor that what was expected (item 20), and one item was excluded from the factor “beliefs about the severity of not exercising” because it incremented the internal reliability from α = 0.53 to α = 0.67 (item 6). The factor structure of the scale reflects each of five the factors of the HBM.

The first factor explained 31.48% of the total variance, and it was composed of six items (items 21 to 26) with factor loadings ranging from 0.41 to 0.92. According to the theme of these items, this factor was named “beliefs that the benefits exceed the costs of exercising”. An item for this factor is “Is it worth paying the price of exercising [such as investing time and overcoming laziness] to prevent diseases in the future?”. The second factor explained 10.52% of the total variance and it consisted of seven items (items 11 to 17) with a range of factor loadings from 0.68 to 0.87. The theme for these items was “beliefs that exercising can reduce threats” and an item from this factor was “How much do you think exercise will help you prevent having high blood pressure?”. The third factor explained 5.97% of the total variance and it included six items (items 27 to 32) with factor loading values between 0.41 and 0.82. The theme for these items was “beliefs about the vulnerability of not exercising” and an item from this factor was “Do you think you could get high blood pressure?”.

The fourth factor explained 5.16% of the total variance, and initially consisted of four items with a factor loadings ranging from 0.31 to 0.91. Item 6 of this factor was excluded because it incremented the Cronbach’s alpha value from 0.53 to 0.67, and the factor ended up consisting of three items (items seven to nine) with factor loadings ranging from 0.45 to 0.91. This factor was named “beliefs about the severity of not exercising” according to the items. An example item is “How serious is it to suffer a heart attack?”. The fifth factor explained 4.34% of the total variance and it was composed of three items (items one to three) with factor loadings ranging from 0.79 to 0.85. The theme of the items was “general health values” and an example item is “How much do you care about your health?”.

### Internal reliability

The internal reliabilities of each factor was calculated with the Cronbach’s alpha index. The factor of “general health values” obtained an internal reliability of α = 0.88; the factor of “beliefs about the vulnerability of not exercising”, α = 0.76; the factor of “beliefs about the severity of not exercising”, α = 0.67; the factor of “beliefs that exercising can reduce threats” α = 0.91; and the factor of “beliefs that the benefits exceed the costs of exercising”, α = 0.82. The internal consistency for all of the items on the scale was not analyzed since the HBM does not include a global index.

## Discussion

The factor structure of the EHBMS was validated in a Mexican sample in our study and it can be used to assess the HBM to explain exercise. This was the first scale validated in Mexico using the HBM applied to the health behavior of exercise. The scale is composed of the 5 components (Fig. 1) of the HBM [33] and most of them have adequate internal reliability coefficients, except for the factor of “beliefs about the severity of not exercising” with α = 0.67.

Mexico has one of the highest percentages of overweight and obesity in adults in the whole world [2], and the development of this scale will help explain a behavior that can be changed. According to the National Institutes of Health, exercise is not the only factor related to overweight and obesity, there are other factors that influence them like the environment, genes, family history, health conditions, medicines, emotional factors, smoking, age, pregnancy and lack of sleep [50], but exercise behaviors have been associated with a reduction of weight and body mass index in several studies [9–14], and this study provides an exercise scale that can measure and analyze a validated model, the HBM, in a Mexican population since the sample used in this study consisted of five different sites across Mexico. The large sample size obtained from the different sites allows for greater generalization of the results based on this scale since the sample was obtained from the north and central part of Mexico.

This scale was based in the HBM and it will be useful for the explanation of exercise, helping health providers promote exercise. These interventions will give people with overweight obesity tools to help them exercise in order to have a better health reducing the risks like suffering from heart attacks or strokes, diabetes or some types of cancer.

It is important to note that there is a need to replicate these findings with other representative Mexican samples from other states, other ages, and other ethnic groups that are part of the country. There is also a need to confirm the factor structure using confirmatory factor analysis with a different sample.

## Conclusions

The Exercise Health Belief Model Scale can be used in Mexican populations to measure the Health Belief Model applied to exercise. The scale has a good factor structure that reflects all of the components of the HBM and it also has acceptable internal reliability indices.

## Abbreviations

EFA: Exploratory factor analysis
EHBMS: Exercise health belief model scale
HBM: Health belief model
KMO: Kaiser-Meyer-Olkin
α: Cronbach’s alpha

## References

[1] World Health Organization: Obesity and overweight. 2016. http://www.who.int/mediacentre/factsheets/fs311/en/. Accessed 15 Sep 2016.

[2] Instituto Nacional de Salud Pública [National Institute of Public Health]: Encuesta nacional de salud y nutrición. Resultados nacionales [National survey of health and nutrition. National results]. 2012. http://ensanut.insp.mx/informes/ENSANUT2012ResultadosNacionales.pdf. Accessed 10 Sep 2016.

[3] Lin X, Zhang X, Guo J, Roberts CK, McKensie S, Wu W, Liu S, Song Y. Effects of exercise training on cardiorespiratory fitness and biomarkers of cardiometabolic health: A systematic review and meta-analysis of randomized controlled trials. J Am Heart Assoc. 2015. doi:10.1161/JAHA.115.002014. 10.1161/JAHA.115.002014 PMC4608087 26116691

[4] Stewart KJ (2002). Exercise training and the cardiovascular consequences of type 2 diabetes and hypertension. Plausible mechanisms for improving cardiovascular health. JAMA.

[5] Batterham SI, Heywood S, Keating JL. Systematic review and meta-analysis comparing land and aquatic exercise for people with hip or knee arthritis on function, mobility and other health outcomes. BMC Musculoskelet Disord. 2011. doi:10.1186/1471-2474-12-123. 10.1186/1471-2474-12-123 PMC3141607 21635746

[6] Kang DW, Lee J, Suh HS, Ligibel JA, Courneya KS, Jeon JY. Effects of exercise on insulin, IGF-axis, adipocytokines, and inflammatory markers in breast cancer survivors: A systematic review and meta-analysis. Cancer Epidemiol Biomarkers Prev. 2016. doi:10.1158/1055-9965.EPI-16-0602. 10.1158/1055-9965.EPI-16-0602 27742668

[7] Mishra SI, Scherer RW, Snyder C, Geigle P, Gotay C. Are exercise programs effective for improving health-related quality of life among cancer survivors? A systematic review and meta-analysis. Oncol Nurs Forum. 2014. doi:10.1188/14.ONF.E326-E342. 10.1188/14.ONF.E326-E342 PMC4332787 25355029

[8] Smith JJ, Eather N, Morgan PJ, Plotnikoff RC, Faigenbaum AD, Lubans DR. The health benefits of muscular fitness for children and adolescents: A systematic review and meta-analysis. Sports Med. 2014. doi:10.1007/s40279-014-0196-4. 10.1007/s40279-014-0196-4 24788950

[9] Rock CL, Flatt SW, Byers TE, Colditz GA, Demark-Wahnefried W, Ganz PA, Wolin KY, Elias A, Krontiras H, Liu J, Naughton M, Pakiz B, Parker BA, Sedjo RL, Wyatt H. Results of the Exercise and Nutrition to Enhance Recovery and Good Health for You (ENERGY) Trial: A behavioral weight loss intervention in overweight or obese breast cancer survivors. J Clin Oncol. 2015. doi:10.1200/JCO.2015.61.1095. 10.1200/JCO.2015.61.1095 PMC4582146 26282657

[10] Williams DM, Dunsiger S, Miranda R Jr, Gwaltney CJ, Emerson JA, Monti PM, Parisi AF. Recommending self-paced exercise among overweight and obese adults: a randomized pilot study. Ann Behav Med. 2015. doi:10.1007/s12160-014-9642-7. 10.1007/s12160-014-9642-7 PMC4355095 25223963

[11] Ruotsalainen H, Kyngäs H, Tammelin T, Kääriäinen M. Systematic review of physical activity and exercise interventions on body mass indices, subsequent physical activity and psychological symptoms in overweight and obese adolescents. J Adv Nurs. 2015. doi:10.1111/jan.12696. 10.1111/jan.12696 26031309

[12] Kelley GA, Kelley KS. Evidential value that exercise improves BMI z-score in overweight and obese children and adolescents. Biomed Res Int. 2015. doi:10.1155/2015/151985. 10.1155/2015/151985 PMC4609764 26509145

[13] Stoner L, Rowlands D, Morrison A, Credeur D, Hamlin M, Gaffney K, Lambrick D, Matheson A. Efficacy of exercise intervention for weight loss in overweight and obese adolescents: Meta-analysis and implications. Sports Med. 2016. doi:10.1007/s40279-016-0537-6. 10.1007/s40279-016-0537-6 27139723

[14] García-Hermoso A, Cerrillo-Urbina AJ, Herrera-Valenzuela T, Cristi-Montero C, Saavedra JM, Martínez-Vizcaíno V. Is high-intensity interval training more effective on improving cardiometabolic risk and aerobic capacity than other forms of exercise in overweight and obese youth? A meta-analysis. Obes Rev. 2016. doi:10.1111/obr.12395. 10.1111/obr.12395 26948135

[15] World Health Organization: Health education: theoretical concepts, effective strategies and core competencies. 2012. http://applications.emro.who.int/dsaf/EMRPUB_2012_EN_1362.pdf. Accessed 3 Jan 2017.

[16] Cabrera G, Tascón J, Lucumí D (2001). Creencias en salud: historia, constructos y aportes al modelo [Beliefs in health: History, constructs and input to the model]. Rev Fac Nac Salud Pública.

[17] Al-Ali N, Haddad LG. The effect of the health belief model in explaining exercise participation among Jordanian myocardial infarction patients. J Transcult Nurs. 2004. doi:10.1177/1043659603262484. 10.1177/1043659603262484 15070493

[18] Lo SW, Chair SY, Lee FK. Factors associated with health-promoting behavior of people with or at high risk of metabolic syndrome: Based on the health belief model. Appl Nurs Res. 2015. doi:10.1016/j.apnr.2014.11.001. 10.1016/j.apnr.2014.11.001 25540911

[19] Mirotznik J, Feldman L, Stein R (1995). The health belief model and adherence with a community center-based, supervised coronary heart disease exercise program. J Community Health.

[20] Soleymanian A, Niknami S, Hajizadeh E, Shojaeizadeh D, Montazeri A. Development and validation of a health belief model based instrument for measuring factors influencing exercise behaviors to prevent osteoporosis in pre-menopausal women (HOPE). BMC Musculoskelet Disord. 2014. doi:10.1186/1471-2474-15-61. 10.1186/1471-2474-15-61 PMC3996026 24581300

[21] Rezapour B, Mostafavi F, Khalkhali H. “Theory Based Health Education: Application of Health Belief Model for Iranian Obese and Overweight Students about Physical Activity” in Urmia, Iran. Int J Prev Med. 2016. doi:10.4103/2008-7802.191879. 10.4103/2008-7802.191879 PMC5070032 27761217

[22] Hoseini H, Maleki F, Moeini M, Sharifirad GR (2014). Investigating the effect of an education plan based on the health belief model on the physical activity of women who are at risk for hypertension. Iran J Nurs Midwifery Res.

[23] King KA, Vidourek RA, Merianos AL. Vigorous physical activity among college students: using the health belief model to assess involvement and social support. Arch Exerc Health Dis. 2014. doi:10.5628/aehd.v4i2.153.

[24] Caspersen CJ, Powell KE, Christenson GM (1985). Physical activity, exercise, and physical fitness: definitions and distinctions for health-related research. Public Health Rep.

[25] Hernández H. Modelo de Creencias en Salud y Obesidad. Un Estudio de los Adolescentes de la Provincia de Guadalajara [Health Belief Model and obesity: A study of adolscents from the province of Guadalajara]. PhD thesis. Universidad de Alcalá, Educational Psychology and Physical Education Department; 2010.

[26] Glanz K, Rimer BK, Lewis FM (2002). Health behavior and health education theory, research and practice.

[27] Hochbaum G (1958). Public participation medical screening programs: A sociological psychological study.

[28] Rosenstock IM (1966). Why people use health services. Milbank Mem Fund Q.

[29] Moreno E, Roales-Nieto J (2003). El modelo de creencias de salud: Revisión teórica, consideración crítica y propuesta alternativa I: Hacia un análisis funcional de las creencias en salud [The Health Belief Model:Ttheoretical review, critical consideration and alternative proposal I: Towards a functional analysis of the beliefs in health]. Int J Psychol Psychol Ther.

[30] Rosenstock IM. Historical origins of the health belief model. Health Educ Behav. 1974. doi:10.1177/109019817400200403.

[31] Janz NK, Becker MH (1984). The Health Belief Model: A decade later. Health Educ Q.

[32] Soto F, Lacoste J, Papenfuss R, Gutiérrez A (1997). El modelo de creencias en salud. Un enfoque teórico para la prevención del SIDA. Rev Esp Salud Publica.

[33] Taylor SE (2007). Health psychology.

[34] Abraham C, Sheeran P, Conner M, Norman P (2005). The health belief model. Predicting and changing health behavior: Research and practice with social cognition models.

[35] Tola HH, Shojaeizadeh D, Garmaroudi G, Yekaninejad MS, Kebede A, Ejeta LT, Kassa D, Klinkenberg E. Psychological and educational intervention to improve tuberculosis treatment adherence in Ethiopia based on Health Belief Model: A cluster randomized control trial. PLoS One. 2016. doi:10.1371/journal.pone.0155147. 10.1371/journal.pone.0155147 PMC4864292 27167378

[36] Abolfotouh MA, BaniMustafa AA, Mahfouz AA, Al-Assiri MH, Al-Juhani AF, Alaskar AS. Using the health belief model to predict breast self examination among Saudi women. BMC Public Health. 2015. doi: 10.1186/s12889-015-2510-y. 10.1186/s12889-015-2510-y PMC4657230 26596507

[37] Jeihooni AK, Hidarnia A, Kaveh MH, Hajizadeh E, Askari A. The Effect of an educational program based on Health Belief Model on preventing osteoporosis in women. Int J Prev Med. 2015. doi:10.4103/2008-7802.170429. 10.4103/2008-7802.170429 PMC4689100 26730345

[38] Bayu H, Berhe Y, Mulat A, Alemu A. Cervical cancer screening service uptake and associated factors among age eligible women in Mekelle Zone, Northern Ethiopia, 2015: A community based study using Health Belief Model. PLoS ONE. 2016. doi:10.1371/journal.pone.0149908. 10.1371/journal.pone.0149908 PMC4786115 26963098

[39] Reynolds GL, Nguyen HH, Fisher DG, Odell A, Xandre P. Application of the Extended Health Control Belief Model to Predict Hepatitis A and B Vaccinations. Public Health Nurs. 2016. doi:10.1111/phn.12254. 10.1111/phn.12254 26918304

[40] Pirzadeh A (2012). The effect of education on women’s practice based on the Health Belief Model about Pap smear test. Int J Prev Med.

[41] Guvenc G, Seven M, Akyuz A. Health Belief Model Scale for Human Papilloma Virus and its vaccination: Adaptation and psychometric testing. J Pediatr Adolesc Gynecol. 2016. doi:10.1016/j.jpag.2015.09.007. 10.1016/j.jpag.2015.09.007 26409648

[42] Zare M, Ghodsbin F, Jahanbin I, Ariafar A, Keshavarzi S, Izadi T (2016). The effect of Health Belief Model-based education on knowledge and prostate cancer screening behaviors: A randomized controlled trial. Int J Community Based Nurs Midwifery.

[43] Wong RK, Wong ML, Chan YH, Feng Z, Wai CT, Yeoh KG. Gender differences in predictors of colorectal cancer screening uptake: a national cross sectional study based on the health belief model. BMC Public Health. 2013. doi:10.1186/1471-2458-13-677. 10.1186/1471-2458-13-677 PMC3726512 23879593

[44] Li X, Lei Y, Wang H, He G, Williams AB. The Health Belief Model: A qualitative study to understand high-risk sexual behavior in Chinese men who have sex with men. J Assoc Nurses AIDS Care. 2016. doi:10.1016/j.jana.2015.10.005. 10.1016/j.jana.2015.10.005 26604043

[45] Mo PKH, Chong ESK, Mak WWS, Wong SYS, Lau TF. Physical Activity in People With Mental Illness in Hong Kong: Application of the Health Belief Model. J Sport Exerc Psychol. 2016. doi:10.1123/jsep.2015-0061. 10.1123/jsep.2015-0061 27390136

[46] Parsa P, Kandiah M, Mohd Nasir MT, Hejar AR, Nor Afiah MZ (2008). Reliability and validity of Champion’s Health Belief Model Scale for breast cancer screening among Malaysian women. Singapore Med J.

[47] Nejad LM, Wertheim EH, Greenwood KM (2005). Comparison of the Health Belief Model and the Theory of Planned Behavior in the prediction of dieting and fasting behavior. E J Appl Psychol.

[48] MacCallum RC, Widaman KF, Keith F, Zhang S (1999). Sample size in factor analysis. Psychol Methods.

[49] Snedecor GW, Cochran WG (1989). Statistical Methods.

[50] National Institutes of Health. What causes overweight and obesity? https://www.nhlbi.nih.gov/health/health-topics/topics/obe/causes. Accessed 4 Jan 2017.

